# Poverty, Wealth, and Health Care Utilization: A Geographic Assessment

**DOI:** 10.1007/s11524-012-9689-3

**Published:** 2012-05-08

**Authors:** Richard A. Cooper, Matthew A. Cooper, Emily L. McGinley, Xiaolin Fan, J. Thomas Rosenthal

**Affiliations:** 1 Department of Medicine and Leonard Davis Institute of Health Economics, University of Pennsylvania, Philadelphia, PA USA; 2 Institute for Health and Society, Medical College of Wisconsin, Milwaukee, WI USA; 3 Department of Urology, University of California, Los Angeles, Los Angeles, CA USA; 4 University of Pennsylvania, Philadelphia, PA USA; 5 New York Institute of Technology, New York, NY USA

**Keywords:** Poverty, Urban, Health care, Geographic variation

## Abstract

Geographic variation has been of interest to both health planners and social epidemiologists. However, while the major focus of interest of planners has been on variation in *health care spending*, social epidemiologists have focused on *health*; and while social epidemiologists have observed strong associations between poor health and poverty, planners have concluded that income is not an important determinant of variation in spending. These different conclusions stem, at least in part, from differences in approach. Health planners have generally studied variation among large regions, such as states, counties, or hospital referral regions (HRRs), while epidemiologists have tended to study local areas, such as ZIP codes and census tracts. To better understand the basis for geographic variation in hospital utilization, we drew upon both approaches. Counties and HRRs were disaggregated into their constituent ZIP codes and census tracts and examined the interrelationships between income, disability, and hospital utilization that were examined at both the regional and local levels, using statistical and geomapping tools. Our studies centered on the Milwaukee and Los Angeles HRRs, where per capita health care utilization has been greater than elsewhere in their states. We compared Milwaukee to other HRRs in Wisconsin and Los Angeles to the other populous counties of California and to a region in California of comparable size and diversity, stretching from San Francisco to Sacramento (termed “San-Framento”). When studied at the ZIP code level, we found steep, curvilinear relationships between lower income and both increased hospital utilization and increasing percentages of individuals reporting disabilities. These associations were also evident on geomaps. They were strongest among populations of working-age adults but weaker among seniors, for whom income proved to be a poor proxy for poverty and whose residential locations deviated from the major underlying income patterns. Among working-age adults, virtually all of the excess utilization in Milwaukee was attributable to very high utilization in Milwaukee’s segregated “poverty corridor.” Similarly, the greater rate of hospital use in Los Angeles than in San-Framento could be explained by proportionately more low-income ZIP codes in Los Angeles and fewer in San-Framento. Indeed, when only high-income ZIP codes were assessed, there was little variation in hospital utilization among California’s 18 most populous counties. We estimated that had utilization within each region been at the rate of its high-income ZIP codes, overall utilization would have been 35 % less among working-age adults and 20 % less among seniors. These studies reveal the importance of disaggregating large geographic units into their constituent ZIP codes in order to understand variation in health care utilization among them. They demonstrate the strong association between low ZIP code income and both higher percentages of disability and greater hospital utilization. And they suggest that, given the large contribution of the poorest neighborhoods to aggregate utilization, it will be difficult to curb the growth of health care spending without addressing the underlying social determinants of health.

## Introduction

The test of our progress is not whether we add more to the abundance of those who have much. It is whether we provide enough for those who have too little.

Franklin D. Roosevelt, 1937

Geographic variation in health care has played a prominent role in shaping health care reform.^1^–^5^ It also has been a subject of interest to social epidemiologists.^6^–^9^ However, there are important differences. First, the focus of interest among planners has been on variation in *health care spending*, while epidemiologists have focused principally on *health*. In addition, while planners have attributed some of the variation to differences in patients’ burden of disease, they have attributed little to income, and much remains “unexplained.”^10^–^15^ In contrast, a broad body of epidemiological literature links low income to poor health and shorter life-spans.^6^–^8^,^16^–^23^ From the perspective of social epidemiologists, poverty has a crushing effect on health.^7^,^23^

Why has poverty been so prominent in epidemiological studies and so out of view in studies of health care spending? One reason is that epidemiologists generally examine data at the level of individuals or within units more reflective of neighborhoods, such as census tracts or postal codes.^24^–^26^ In contrast, health planners have generally studied much larger units, such as counties, hospital referral regions (HRRs), or states.^10^,^12^,^15^,^27^ Aggregating populations in units as large and diverse as these has tended to blur the effects of social factors that are so readily apparent in units of smaller size.^28^–^31^ As Krieger has warned, “Blot poverty from view and not only will we contribute to making suffering invisible but our understanding of disease etiology will be marred.”^7^

We have attempted to gain insight into the basis for geographic variation in health care among larger units by disaggregating them into their constituent ZIP codes and census tracts and assessing hospital utilization, household income, and the prevalence of disability, both statistically and spatially. Our studies centered on two urban HRRs, Milwaukee and Los Angeles. The Milwaukee HRR is not only the most populace in Wisconsin but also the most racially and economically segregated, and it utilizes more health care per capita than other HRRs in the upper Midwest.^27^,^32^,^33^ The Los Angeles HRR is the most populous in the nation, and its rate of health care utilization is among the nation’s highest.^34^ We compared Milwaukee to other Wisconsin HRRs and Los Angeles both to a region of comparable size, stretching from San Francisco to Sacramento (termed “San-Framento”), and other populace California counties. These studies revealed the profound contribution of the poorest ZIP codes of each region to geographic variation in health care utilization among regions.

## Methods

ZIP code-level hospital data for Wisconsin were obtained from the Person-Level Data and Analysis Section of the State of Wisconsin Bureau of Health Information and were averaged for the years 1999 through 2002. The Milwaukee HRR was compared with seven others in Wisconsin: Appleton, Green Bay, La Crosse, Madison, Marshfield, Neenah, and Wausau. ZIP code level hospital data for California were obtained from the Patient Discharge Data File of the Office of Statewide Health Care Planning of the State of California for the year 2008. The Los Angeles region consisted of Los Angeles County, which overlaps the Los Angeles HRR. The San-Framento region encompassed San Francisco, Marin, San Mateo, Santa Cruz, Alameda, Contra Costa, Santa Clara, San Joaquin, Solano, and Sacramento counties. In both states, measurements of inpatient hospital days were limited to adults in acute care hospitals, exclusive of admissions related to pregnancy and child-birth. Admissions to skilled nursing, intermediate care, psychiatric, chemical dependency, and physical rehabilitation facilities were excluded. Only the Wisconsin and California portions of HRRs that extended into adjacent states were analyzed.

Population and income data at the ZIP code level were from the Census Bureau, either directly or through other sources.^35^ For studies in Wisconsin, census data for 2000 were also extracted from GeoLytics Professional (GeoLytics, Inc., East Brunswick, NJ). For studies in California, estimates for 2008 were obtained from Claritas PopFacts (Tetrad Computer Applications, Inc., Ferndale, WA). Data on poverty and disability from all causes by age were from the 2000 census, as complied by GeoLytics.

ZIP codes were excluded where the principal populations were university students, military personnel, or institutionalized populations or where the total adult population was less than 1,500. The final analyses included 107 ZIP codes in Milwaukee, 266 in Los Angeles, and 287 in San-Framento. The total adult population included was 1.44 million in Milwaukee, 7.48 million in Los Angeles, and 6.94 million in San-Framento. Data were mapped at the ZIP code level using Mapland Professional (Software Illustrated, Tracy CA) and at the census tract level using GeoLytics Long Form. Goodness of fit was calculated using the power trend function of Microsoft PowerPoint. Pearson correlation coefficients were calculated using the statistical tool of Microsoft Excel.

## Results

### Milwaukee

The Milwaukee HRR includes both the city of Milwaukee and a surrounding zone ten times as large in area but roughly equivalent in population. Health care spending in the Milwaukee HRR exceeds the rate in other parts of the upper-Midwest by approximately one third,^27^ a fact that has concerned Milwaukee’s business community.^32^

**Figure 1.**
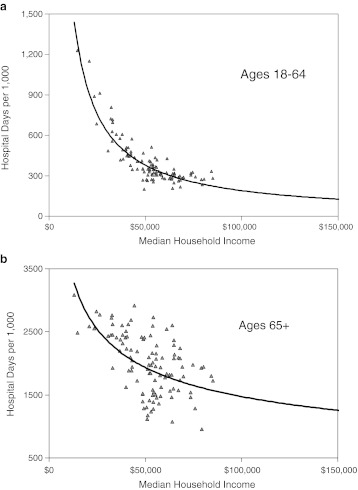
Median household income (MHI) and hospital days in Milwaukee. The MHIs of ZIP codes in the Milwaukee hospital referral region (HRR) were compared with the numbers of hospital days per 1,000 of population. Power regression. **a** Working-age adults (ages 18–64). **b** Seniors (ages 65+).

We divided Milwaukee’s adult population into working-age adults (ages 18 to 64) and seniors (ages 65 and over). Our initial studies were carried out among the former. When assessed at the ZIP code level, there were steeply inverse, curvilinear relationship between median household income (MHI) and both the number of hospital days per 1,000 (*r* ^2^ = 0.755; Figure 1a) and the per cent of the ZIP code population reporting a disability (*r* ^2^ = 0.636). The relationship between MHI and hospital days could also be resolved into two linear components: one at household incomes below $50,000 (39 % of ZIP codes), which had a steep slope and strong coefficient (*r* ^2^ = 0.818), and the other at incomes above $50,000, which was relatively flat. Utilization was 3-fold greater in ZIP codes comprising the lowest income decile vs. the highest

**Figure 2.**
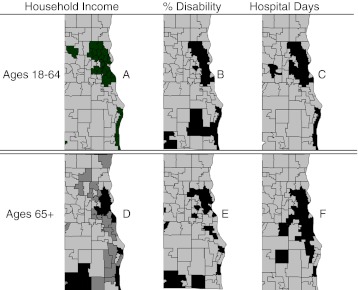
Geomapping household income, disability, and hospital days in Milwaukee. *Areas shaded black* are the quintiles of ZIP codes with the least MHI (*A*, *D*), the most disability (*B*, *E*) and most hospital days per 1,000 (*C*, *F*). The *area shaded dark gray* in panel *D* is the next lowest quintile of MHI. The region shown includes the portion of the Milwaukee HRR with the highest population density.

These statistical relationships were also evident in geomaps (Figure 2). The spatial distribution of the quintile of ZIP codes with the lowest MHIs (A) was similar to those with the highest percent of disability (B), and these were similar to those with the most hospital days per 1,000 of population (C).

Much of the increase in utilization in low-income ZIP codes was due to admissions for ambulatory care-sensitive conditions. Comparing utilization among adults ages 35–64 in the lowest vs. the highest income quartile of ZIP codes in Milwaukee County, the number of hospital days per 1,000 was greater by 347 % for heart failure, 266 % for diabetes, and 610 % for chronic obstructive pulmonary disease (COPD), increments similar to those observed previously in other urban areas.^36^–^38^

#### Seniors

Seniors accounted for 17 % of the adult population but utilized 49 % of the total number hospital days. As observed among working-age adults, there were significant associations between MHI and both hospital utilization and disability among seniors, but these were weaker than among working-age adults. Accordingly, when hospital utilization was plotted against MHI (Figure 1b), the data were more scattered (*r* ^2^ = 0.304) and the amplitude of differences in utilization between the poorest and richest ZIP codes was less than had been observed for working-age adults. ZIP code maps confirmed these statistical differences (Figure 2).

Two factors appeared to contribute to these differences between seniors and working-age adults. One was a difference in residential distribution. Census tract maps of seniors showed many low-income tracts within higher-income ZIP codes. Similarly, ZIP code maps showed the presence of low-income seniors in areas in which higher income working-age adults resided (Figure 2A, D). This was due, in part, to the distribution of nursing homes and senior housing. Indeed, Milwaukee’s poverty core is devoid of nursing homes. Conversely, census tract maps showed clusters of high-income seniors in predominantly low-income ZIP codes, corresponding to the locations of luxury apartments in the central city. Thus, the high degree of income segregation that exists among working-age adults does not continue beyond age 65, and the patterns of hospital utilization followed accordingly.

**Figure 3.**
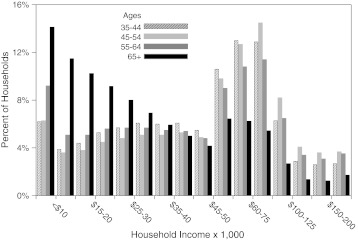
Household income among age groups. The distribution of MHI in 2008 is displayed for ages 35–44 (*hatched*), 45–54 (*light gray*), 55–64 (*dark gray*), and 65+ (*black*).

A second factor is a difference in income distribution among seniors and working-age adults (Figure 3). During the decades of working life, incomes are skewed to higher incomes, whereas after age 65, incomes are sharply skewed to low income. Some low-income seniors were poor earlier in life and, therefore, may have experienced chronic poverty,^39^–^41^ while others became low income in retirement but had the advantages of higher income in earlier decades. This phenomenon decreases the validity of low income as a proxy for poverty as it relates to health care utilization among seniors.

#### Milwaukee’s Poverty Corridor

**Figure 4.**
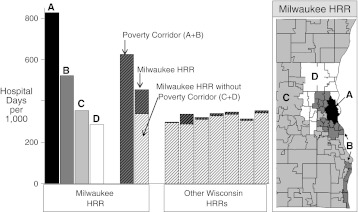
Milwaukee HRR zones and Wisconsin HRRs. The *four bars on the left* illustrate the utilization of hospital days per 1,000 among 18–64 year olds in the ZIP code zones illustrated in the figure on the *right*: *A* poverty core, *B* remainder of poverty corridor, *D* affluent rim, *C* remainder of Milwaukee HRR. The *next two bars* illustrate utilization in the poverty corridor (*dark hatched*) and in the entire Milwaukee HRR, including the poverty corridor (the *dark hatched section*). The *final seven bars* illustrate utilization in other Wisconsin HRRs and in the ZIP codes of each with MHIs <$40,000 (*dark hatched*).

Because of Milwaukee’s extreme racial and economic segregation, we were able to define a narrow “poverty corridor” (Figure 4A + B) in which the MHI was 40 % lower than elsewhere in Milwaukee. The corridor included 41 % of the adult population but 85 % of the black and Hispanic populations, and blacks and Hispanics residing there accounted for more than one third of the population, as compared to fewer than 5 % elsewhere. In the core area of extreme poverty (A), blacks and Hispanics comprised more than two thirds of the population and the poverty rate was 72 %. Hospital utilization among working-age adults was 85 % greater in the poverty corridor (A + B) than in the remainder of the Milwaukee HRR (C + D), and it was 145 % greater in the core area of greatest poverty (A).

Compared to other HRRs in Wisconsin, hospital utilization among working-age adults in Milwaukee was 38 % greater. However, when both Milwaukee’s poverty corridor and the poorest ZIP codes of other HRRs were excluded, the difference decreased to 5.4 %. Because low-income seniors were distributed more widely (Figure 2C), excluding the poverty corridor had less effect on their utilization, but even among seniors, removing the corridor from consideration reduced the difference in utilization between Milwaukee and other Wisconsin HRRs from 44 % to 29 %. Thus among working-age adults, the poverty corridor accounted for almost all of the difference in utilization between Milwaukee and other HRRs, and it accounted for almost half among seniors.

The highest-income area of Milwaukee was a rim of ZIP codes that capped the poverty corridor (Figure 4D). Compared to this affluent rim, utilization in the poverty corridor was more than double among working-age adults and one-third greater among seniors. If the utilization of health care throughout Milwaukee had been at the rate of the affluent rim, the number of hospital days per 1,000 would have been 37 % less among working-age adults, 13 % less among seniors, and 25 % less overall (Table 1). Thus, the poorest ZIP codes in Milwaukee were the major contributors to higher hospital utilization in the Milwaukee HRR.

**Table 1:** Incremental utilization of hospital days above “affluent standard”

	Ages	Ages	Ages	Ages	All
	18–64	18–44	45–64	65+	ages
**Milwaukee**					
At rate of Affluent Rim	37.2 %			13.0 %	25.2 %
At rate of ZIP code incomes >$60,000	35.9 %			10.9 %	26.7 %

**Los Angeles**					
At rate of ZIP code incomes >$75,000	44.7 %	35.3 %	45.0 %	30.7 %	37.2 %

**San-Framento**					
At rate of ZIP code incomes >$75,000	26.0 %	22.0 %	33.1 %	11.2 %	18.1 %

**7 California Counties** ^a^					
At rate of ZIP code incomes >$75,000	21.8 %	16.8 %	31.4 %	10.6 %	15.8 %

**18 California Counties** ^b^					
At rate of ZIP code incomes >$75,000	28.3 %	23.7 %	36.6 %	19.3 %	26.3 %

^a^California counties in Figure 7, excluding Los Angeles

^b^Populace counties that include 85 % of California's adult population

### Los Angeles

Because Los Angeles is so populous, we were able to study two cohorts of working-age adults, ages 18–44 and 45–64, and third cohort of seniors. Of these, the 45–64-year-old cohort displayed the strongest relationships between hospital utilization both income and disability, and the analyses that follow focus on this cohort.

#### Statistical Relationships

**Figure 5.**
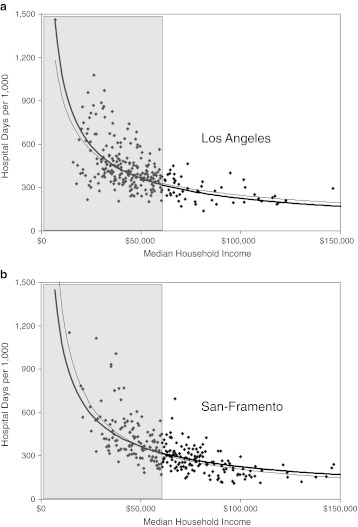
Median household income and hospital days in Los Angeles and San-Framento. The MHIs of ZIP codes were compared with the numbers of hospital days per 1,000 of population among ages 45–64 in Los Angeles (**a**) and San-Framento (**b**). Power regressions. The *heavy line* in both is the regression derived from the combined regions. The *shaded area* distinguishes ZIP codes with MHIs <$60,000.

Like Milwaukee, Los Angeles has both affluent and poor areas, but unlike Milwaukee, where poverty is largely confined to a narrow corridor, poverty exists both in a central core and in scattered clusters elsewhere, many adjacent to affluent neighborhoods. Nonetheless, as in Milwaukee, there was a steeply inverse, curvilinear relationships between hospital days per 1,000 and MHI (*r* ^2^ = 0.440; Figure 5a). The magnitude of difference in utilization between ZIP codes containing the poorest and wealthiest deciles of the population was almost 3-fold.

The 18–44-year-old cohort utilized approximately one third as many hospital days per 1,000 as the 45–64-year-old cohort, and accordingly the statistical relationship was weaker (*r* ^2^ = 0.259), but the range of difference in utilization between the wealthiest and poorest ZIP codes was the same in both cohorts. Across both age cohorts, there was a strong relationship between the MHI of ZIP codes and the percent disabilities, which best fit a power function (*r* ^2^ = 0.613). As in Milwaukee, increases in hospital utilization in low-income ZIP codes could be partially explained by higher rates of admission for ambulatory care sensitive conditions, which across all adult ages were twice as frequent in the area of highest poverty as in the other areas of Los Angeles.^42^

#### Seniors

The relationship between income and hospital utilization was weakest among seniors (*r* ^2^ = 0.220), as also observed in Milwaukee, and the magnitude of difference in utilization between the poorest and wealthiest deciles was half as great. When viewed on geomaps, both poverty and high rates of hospital utilization were distributed more broadly among seniors than among working-age adults. Reflecting this wider distribution, the ratio of seniors to 45–64 year olds in the lowest-income quintile was 20 % lower than in the highest. Finally, to an even greater degree than in Milwaukee, there were clusters of seniors in high-income census tracts within predominantly low-income ZIP codes in central Los Angeles. Thus, as in Milwaukee, ZIP codes proved to have less fidelity for assessing the economic characteristics of seniors than of working-age adults.

#### Mapping Los Angeles

**Figure 6.**
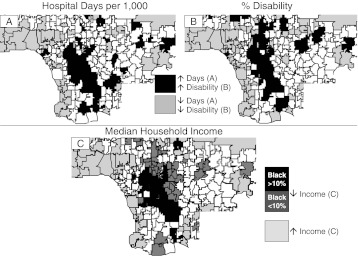
Geomapping hospital days and poverty in Los Angeles. The *areas shaded black* or *dark gray* are the quartiles of ZIP codes with most hospital days per 1,000 (**a**), the highest percentages of disability (**b**), and the lowest median household income (**c**), while the areas *lightly shaded* have the lowest hospital utilization, lowest % disability and highest income. In (**c**), the area of low income in with >10 % blacks is shaded *black*, while the area with <10 % blacks is shaded *dark gray*.

Figure 6 displays ZIP codes that encompass quartiles of the 45–64-year-old population with the highest and the lowest income, the highest and lowest per cent disability, and highest and lowest rates of hospitalization utilization. The average rate of utilization in the quartile with the highest was 3.0-fold that of the lowest (Figure 6a) and MHI in the highest was 3.2-fold the lowest (Figure 6c). There was strong overlap between areas of high-income, low-disability, and low-hospital utilization. The converse was also true, with strong overlap between areas of low income, high percentages of disability, and high rates of hospital utilization. However, the overlap with low income (Figure 6c) was greatest for ZIP codes in which there were more than 10 % blacks (average = 30 %), while a group of low-income ZIP codes with fewer than 10 % blacks fell outside of zone of highest utilization.

#### Impact of Low-Income ZIP Codes

To assess the contribution of low-income ZIP codes to overall hospital utilization, we calculated the number of hospital days that would have been utilized in Los Angeles if the rates of utilization in all ZIP codes had been at the rate of those with MHIs above $75,000 (mean = $96,600), which included 5 % of the population. Had utilization everywhere been at this rate, it would have been 45 % less among 45–64 year olds, 31 % less among seniors and 37 % less overall (Table 1).

Thus, although separated by 2,000 miles and with populations that differed by a factor of five, the Milwaukee and Los Angeles HRRs proved to be more similar than different. Both had high degrees of income inequality; both had high rates of hospital utilization; and in both, the patterns of utilization followed underlying income differences, with the highest utilization in areas of greatest poverty and disability and the lowest in areas of greatest wealth and health.

### San-Framento and California Counties

Because poverty in Los Angeles is not confined to a core area, it was not possible to carve-out a poverty corridor as in Milwaukee. Instead, we compared Los Angeles to a region in northern California (San-Framento) with a similar population but a lower rate of hospital utilization.

San-Framento is a 10-county area stretching from San Francisco to Sacramento. It has 90 % the population of Los Angeles and is principally urban, although its land mass is larger due to farming areas between urban centers. However, the sociodemographic characteristics of these two regions are quite different. San-Framento has fewer Hispanics (20 % vs. 45 % in Los Angeles), more non-Hispanic whites (50 % vs. 31 %) and more Asians (18 % vs. 12 %) but similar percentages of blacks (8 % vs. 9 %). MHI is one-third greater in San-Framento than in Los Angeles, and the poverty rate is one-third lower. Most important in terms of the current study, Medicare enrollees have been reported to use 40 % more hospital days in Los Angeles than in San-Framento,^27^ which is similar to the 39 % difference that we observed among seniors.

#### San-Framento vs. Los Ángeles

Despite these differences in sociodemographic characteristics and hospital utilization, the shapes of the curves relating MHI to hospital utilization were virtually identical in Los Angeles and San-Framento (Figure 5a, b), and the goodness of fit in San-Framento was also similar (*r* ^2^ = 0.545). How do these similarities reconcile with the overall differences in utilization between the Los Angeles and San-Framento?

The explanation emerges from a comparison of Figure 5a, b, which shows data for the 45–64-year-old cohort. While the arcs that define the regressions in each were virtually identical, there were more low-income, high-utilization ZIP codes in Los Angeles (the shaded area in Figure 5a) and more high-income, low-utilization ZIP codes in San-Framento (the non-shaded area in Figure 5b). Across all ZIP codes, utilization among ages 45–64 in Los Angeles was 27 % greater than in San-Framento. However, when only those ZIP codes with MHIs >$75,000 were compared, it was only 4 % greater. Similarly, among 18–44 year olds, utilization across all ZIP codes was 24 % greater in Los Angeles than in San-Framento but only 3 % greater in high-income ZIP codes, and among seniors, these differences were 39 % and 9 %. Thus, differences in aggregate hospital utilization between Los Angeles and San-Framento appear to be due principally to differences in the relative numbers of low-income ZIP codes.

Even though San-Framento had fewer low-income ZIP codes than Los Angeles, these contributed substantially to overall utilization (Table 1). Had utilization throughout San-Framento been at the rate of its highest-income ZIP codes, the overall rate would have been 18 % less, half the decrement in Los Angeles but substantial.

#### California Counties

**Figure 7.**
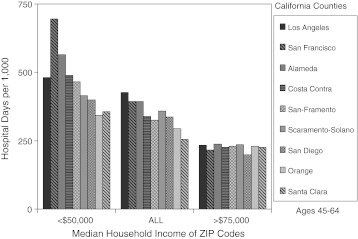
Hospital utilization and median household income in California counties. The set of bar graphs on the *left* illustrates the number of hospital days per 1,000 in low-income ZIP codes (MHIs <$50,000) within eight counties and San-Framento. The *middle set* illustrates utilization in all ZIP codes in these counties, and the set on the *right* illustrates utilization in high-income ZIP codes (MHIs >$75,000.)

Figure 7 extends this analysis to the eight counties in California that have both high-income (MHI >$75,000) and low-income (MHI <$50,000) ZIP codes. When all ZIP codes were considered, the range of variation among counties in the 45–64-year-old cohort was 67 % and the coefficient of variation (COV) was 0.161. When only low-income ZIP codes were considered, the range widened to 103 % and the COV to 0.252; whereas, when only high-income ZIP codes were considered, the range of variation decreased to only 18 % and the COV fell to 0.056. Comparable results were obtained at ages 18–44 (COV = 0.127, 0.226, and 0.082, respectively) and at ages 65+ (COV = 0.153, 0.154, and 0.088). Thus, variation in hospital utilization among counties was strongly influenced by the proportion of low-income ZIP codes. Indeed, there was virtually no variation when only the more affluent ZIP codes were considered.

The contribution of low income to utilization was assessed in the seven counties other than Los Angeles included in Figure 7, as was previously done for Milwaukee, Los Angeles, and San-Framento (Table 1). Had utilization in each of these counties been at the rate of its wealthiest ZIP codes, there would have been 31 % fewer hospital days among 45–64 year olds and 16 % fewer among all adults. We extended this analysis to the 18 most populace California counties (from a total of 59), whose combined adult population of 24.8 million represents 85 % of the total adult population of California. Had utilization in each of these been at the rate of its affluent ZIP codes, there would have been 37 % fewer hospital days among 45–64 year olds and 26 % fewer among all adults (Table 1). Thus, the increased utilization in low-income ZIP codes throughout the most populace counties of California proved to be a major contributor to overall hospital utilization and to account for most of the observed variation in utilization among them.

## Discussion

Four principal conclusions emerge from these studies. First, understanding geographic variation among large regions, such as counties and HRRs, requires disaggregation into their constituent ZIP codes and census tracts. Second, residents of low-income ZIP codes have greatly increased rates of disability and hospital utilization. Third, assessments of the relationship between income and hospital utilization are more valid among working-age adults than among seniors. And finally, poverty varies geographically and its variation explains a great deal about geographic variation in health care utilization. A series of observations contributed to these conclusions:

- In Milwaukee, Los Angeles, and San-Framento, per capita rates of both hospital utilization and disability were steeply increased in ZIP codes with lower MHIs.
- The much higher rates of hospital utilization in Milwaukee as compared with other Wisconsin HRRs were largely explained by the very high rates in Milwaukee’s dense poverty corridor.
- Similarly, the much higher rates of utilization in Los Angeles as compared with San-Framento could be explained by a greater proportion of low-income ZIP codes in Los Angeles and a greater proportion of high-income ZIP codes in San-Framento, while the underlying statistical relationships between income and utilization were the same in both.
- Among eight populace California counties that had both high-income and low-income ZIP codes, the wide variation in utilization that was observed overall was further exaggerated when only low-income ZIP codes were compared but was virtually absent when only high-income ZIP codes were considered.
- In each region, the results of ZIP code analyses were statistically stronger and the impact of low income was quantitatively greater among working-age adults than among seniors.
- These weaker results for seniors appeared to be due both to the wider residential distribution of seniors with respect to income and to weaker associations between low income and chronic poverty among seniors than among working-age adults.
- If hospital utilization within the various regions and counties studied had been at the rate of the high-income ZIP codes in each, it would have been approximately 35 % less among working-age adults, 20 % less among seniors, and 30 % less overall.

Taken together, these studies demonstrate the profound association between poverty and health care utilization.

### Units of Analysis

A central purpose of this study was to analyze data at the micro-level (ZIP codes and census tracts) in order to understand variation in health care utilization among units of macro-size (HRRs and counties) within the broader frameworks of their states.^43^–^45^ We compared the Milwaukee HRR with others in Wisconsin, the Los Angeles HRR with both the “San-Framento” region and other counties in California, and various California counties with each other. By disaggregating these larger units into their constituent ZIP codes, we were able to discern the effects of sociodemographic factors operating at the level of “neighborhoods” on aggregate measures at the regional level.^24^,^25^,^46^–^48^

### Poverty and Geographic Variation

We chose to study Milwaukee and Los Angeles because they have been singled out as high utilization HRRs, Milwaukee in relation to the upper-Midwest and Los Angeles nationally. In these two regions and in San-Framento, we found strong, steeply inverse, curvilinear relationships between income and hospital utilization, reminiscent of the classic relationship between income and mortality,^49^ and similar relationships between income and disability. Among working-age adults, the magnitude of difference between the poorest and richest deciles of population was approximately 3-fold.

Because Milwaukee is so highly segregated, most low-income ZIP codes were clustered in a narrow poverty corridor, which also proved to be the zone of highest hospital utilization. While hospital utilization among working-age adults was one third higher in the Milwaukee HRR than in other HRRs in Wisconsin, utilization in the portion of the Milwaukee HRR outside of the corridor was within 5 % of other Wisconsin HRRs.

Los Angeles presented a greater challenge, but the conclusions were the same. Hospital utilization in Los Angeles was greater than in San-Framento, but this was simply because Los Angeles had a higher proportion of low-income, high-utilization ZIP codes while San-Framento had proportionately fewer, while utilization at comparable levels of income was the same. However, because the regression arcs in Figure 5 transcend differences in utilization of 3-fold and more, many-fold greater than the 25–35 % differences in aggregate utilization between these two regions, small shifts in the proportion of low-income ZIP codes were sufficient to account for the aggregate differences observed. Similarly, variation in hospital utilization among California counties virtually disappeared when only their high-income ZIP codes were considered.

### Seniors

While low income proved to be a strong correlate of hospital utilization among working-age adults, it was a weaker correlate among seniors. Similar discordance between the explanatory power of income in working-age adults and seniors has been noted previously in studies of disease prevalence and mortality, not only in the USA but in Canada, Europe, Japan, and elsewhere.^8^,^50^–^52^

One reason for this discordance, which was apparent on geomaps, was an out-migration of low-income seniors from the poorest ZIP codes into surrounding areas of higher income and, to a lesser extent, an in-migration of wealthy seniors into high-income enclaves within low-income ZIP codes. The former was also inferred from the lower ratio of seniors to 45–64 year olds in low-income ZIP codes than in high-income ones and is accounted for, at least in part, by the location of senior housing and nursing homes. The latter is related to the location luxury apartments within inner-city ZIP codes. These phenomena, which were most apparent at the census tract level, resulted in greater economic heterogeneity at the ZIP code level for seniors than for working-age adults. Previous studies indicate that such movements are not random with respect to health but, rather, that seniors migrating from lower to higher-income areas have higher medical expenditures, while wealthier seniors migrating into lower-income ZIP codes have lower expenditures.^53^ Thus, while ZIP code income appears to provide a valid representation of the economic status of working-age adults, it is a much poorer proxy among seniors.

A second reason for discordance relates to the increase proportion of seniors with low-income as compared with working-age adults. While some of these seniors were poor earlier in life and experienced durable and often multigenerational poverty,^21^–^23^,^39^–^41^ others newly acquired low income after a lifetime of higher income and better health. Assessing poverty has been a challenge at all ages, but it is a particular problem at older ages.^54^–^56^ Indeed, some have suggested that wealth or education may be better indices.^53^,^54^,^57^,^58^

This presents an enigma. While there were strong associations between income and hospital utilization in the 45–64-year-old cohort, these associations were much weaker and of lesser magnitude in the next decade. It seems implausible that such income-related differences would suddenly diminish after age 65. Rather, it is likely that aggregation of dissimilar income groups within ZIP codes and uncertainty over the meaning of low income over age 65 created ambiguities. The aggregation error becomes compounded when ZIP codes are further aggregated into counties or HRRs, further masking income-related differences.^11^–^15^ Yet, it is the Medicare population that has been the principal object of study in defining geographic variation in health care, and it is from such studies that the notion of “unexplained” variation was derived.^10^–^15^,^27^,^59^ Our research suggests that much of this previously “unexplained” variation simply reflects the inability to adequately measure the contribution of low income to health care utilization in the Medicare population, even at the ZIP code level and especially at the level of HRRs.

### Health and Wealth

A wealth of literature has documented the association between low income and poorer health. Parameters such as disease prevalence, disability and mortality have been found to be 2-fold to 3-fold greater in the poorest vs. the richest segments of the population, both in the US and other developed countries.^50^,^60^–^62^ Fewer studies have examined the association between lower income and greater health care utilization, but some exist. These have shown relationships between low income and higher health care expenditures,^14^,^63^–^65^ more hospital admissions,^2^,^66^ more preventable hospitalizations,^36^–^39^,^42^,^67^ and more out-patient visits.^68^,^69^ Low income has also been linked to lower educational attainment, which has separately been shown to correlate with increased disease prevalence, shorter life expectancy, and higher Medicare expenditures.^16^,^49^,^50^,^70^,^71^

Table 1 lists the differences in hospital utilization that would have occurred within various regions if utilization rates in each ZIP code had been at the level of the region’s wealthiest ZIP codes. Taken together, these differences account for approximately 35 % of the total number of hospital days among working-age adults, 20 % among seniors, and 30 % among all adults. The 20 % increment that we observed among seniors is similar to increments in aggregate spending above the expenditure level of high-income Medicare enrollees reported elsewhere.^14^,^63^,^64^ Similarly, the 30 % increment across the entire adult population is similar to increments in hospital admissions, preventable hospitalizations, and expenditures attributable to lower income in previous studies.^65^–^67^ It also is similar to Marmot’s estimate that one third of spending in the British National Health Service (NHS) results from income inequality.^72^

While low-income patients consume more services today, that was not always the case. Forty years ago, they consumed less, both through Medicare and the NHS.^73^–^75^ It was not until the early 1980s that parity was reached, and health care spending among low-income patients has risen disproportionately ever since.^14^,^48^,^53^,^63^,^64^,^76^ Yet, this added spending for the poor is still not viewed as commensurate with their burden of illness,^65^,^77^ and despite it, the gap in life expectancy between rich and poor continues to widen.^70^,^78^

### Limitations

Our studies have several methodological limitations. First, they were derived from studies of predominantly urban regions within Wisconsin and California and may not apply equally to other states or the nation as a whole, although they are consistent with many previous reports of poorer health and greater utilization among the poor. Second, they were confined to adults in acute-care hospitals and may not accurately represent differences in total health care utilization, although the increments in our study are similar to previously reported increments in both inpatient and outpatient care among low-income patients.^36^–^38^,^66^–^69^,^79^

Third is the issue of homogeneity. Census tracts encompass relatively homogeneous populations, but ZIP codes were created for postal routes. While they generally provide valid measures,^24^,^25^,^31^ that ability depends on their homogeneity with respect to the characteristics studied. This proved to be greatest in Milwaukee, one of the most segregated cities in the nation, but wealth and poverty were more comingled in Los Angeles, San-Framento, and elsewhere. In addition, although our studies focused on areas of higher population density, ZIP codes with fewer than 5,000 adults comprised 5 % of all ZIP codes studied in Los Angeles, 9 % in San-Framento, 17 % in other California counties, 25 % in the Milwaukee HRR, and 75 % elsewhere in Wisconsin. The resulting errors were magnified among 18–44 year olds, whose hospital admission rates were low, and among seniors, who account for fewer than 20 % of adults and who are more dispersed relative to income. Thus, although disaggregation of counties and HRRs into ZIP codes resolved many of the errors of aggregation that existed in larger units, the problem persisted even in units as small as ZIP codes.

### Implications

Our studies have several important implications for health and social policy. First, they demonstrate the strong association between poverty and increased health care utilization. This should not be surprising, since poverty and its associated social determinants are known to be linked to poor health status. Our study connects these two realities and documents the large magnitude of added health care utilization that results.

It follows that, since poverty is distributed geographically, geographic differences in health care utilization are largely the result of geographic differences in poverty. That proved to be the case in our studies. Indeed, when only ZIP codes with higher degrees of wealth were considered, there was very little variation at all, which serves to emphasize the need to disaggregate large units of analysis, such as HRRs, if differences in health care utilization among them are to be understood.

Finally, our studies demonstrate that the relationship between poverty and health care utilization, which is so evident among working-age adults, is partially obscured among retirees. This suggests caution in interpreting studies of geographic variation in health care among the Medicare population, which have played such a prominent role in shaping policy.

As the USA seeks to slow the growth of health care spending, it will be important not to conflate the greater amounts of health care utilized by low-income patients with inefficiencies in clinical practice. Even with continued efforts to increase clinical efficiency, it seems unlikely that the inexorable growth in health care spending can abate as long as income inequality continues to widen. The real “inefficiency” is the existence of a population that has not been adequately nurtured in childhood nor given the tools to be healthy adults.^39^–^41^ Poverty is not only an unsustainable failure of social justice. It creates an unsustainable financial burden for our health care system. Accepting this reality is a necessary first step. Confronting it should be our Nation’s highest priority.
